# A Comparability Study Between Intravenous Contrast-Enhanced Cone-Beam Computed Tomography (CBCT) and Magnetic Resonance Angiography (MRA) on the Post-Treatment Follow-Up of Intracranial Aneurysms: A Single-Center Prospective Cohort Study †

**DOI:** 10.3390/diagnostics15141774

**Published:** 2025-07-14

**Authors:** Man Cho Lee, King Him Fung, Shing Him Liu, Koel Wei Sum Ko, Nok Lun Chan, Neeraj Ramesh Mahboobani, Ka Wai Shek, Tak Lap Poon, Wai Lun Poon

**Affiliations:** 1 Queen Elizabeth Hospital, Hong Kong; fkh666@ha.org.hk (K.H.F.); lsh264@ha.org.hk (S.H.L.); koelko@ha.org.hk (K.W.S.K.); ml.chan@ha.org.hk (N.L.C.); skw318@ha.org.hk (K.W.S.); ptl220@ha.org.hk (T.L.P.); poonwl@ha.org.hk (W.L.P.); 2 Prince of Wales Hospital, Hong Kong; neerajmahboobani@cuhk.edu.hk

**Keywords:** cone-beam computed tomography, magnetic resonance angiography, intracranial aneurysm

## Abstract

**Background:** MRA is used in our center for monitoring post-treatment residual aneurysmal neck and stent patency. IV CBCT offers better spatial resolution and may provide significant advantages. **Objective:** This study investigates the image quality of IV CBCT compared to that of MRA for the follow-up of intracranial aneurysms. **Materials and Methods:** In this prospective cohort study, 97 patients (mean age: 63.1 ± 11.7; 75 women and 22 men) with 114 treated cerebral aneurysms were included from July 2023 to April 2024. All patients underwent IV CBCT and MRA on the same day. Two neurointerventional radiologists assessed image quality using a five-point Likert scale on two separate occasions six weeks apart. Diagnostic values were evaluated across six parameters. Intra-observer and inter-observer agreements were calculated. Subgroup analyses were performed. **Results:** Overall, IV CBCT and MRA are comparable in terms of their ability to assess parent vessel status and the degree of artifacts (*p* > 0.05) though MRA shows a slight advantage in evaluating residual aneurysmal neck (*p* = 0.05). For clipped aneurysms, IV CBCT is superior in assessing residual aneurysmal neck (OR = 16.0, *p* < 0.001) and parent vessel status (OR = 15.1, *p* < 0.001) with significantly fewer artifacts (OR > 100, *p* < 0.001). For aneurysms solely treated with stents, IV CBCT is superior in assessing residual aneurysmal neck (OR > 20, *p* = 0.002) and parent vessel status (OR > 20, *p* = 0.002) with significantly fewer artifacts (OR > 20, *p* = 0.002). IV CBCT outperforms MRA in evaluating stent struts and the vessel wall status of a stented segment when MRA is non-diagnostic. **Conclusions:** IV CBCT and MRA have their own strengths and roles in the follow-up of post-treatment intracranial aneurysms. Overall, IV CBCT is superior in terms of its assessment of intracranial aneurysms treated solely with stents or surgical clips.

## 1. Introduction

The management of intracranial aneurysms often involves open surgery such as clipping or endovascular treatments such as coiling and/or stents [1,2,3,4].

Digital subtraction angiography (DSA) remains the gold standard for the detection of residual aneurysm and the assessment of the parent vessel status and stent patency. However, due to its invasiveness, magnetic resonance angiography (MRA) is a valuable alternative where the utilization of different sequences has led to improved image quality over the past few decades [5,6,7]. Conventional multidetector computed tomography angiography (CTA) is not the preferred option due to the presence of significant metallic artifacts.

In recent years, cone-beam computed tomography angiography with intravenous contrast (IV CBCT) has become promising for the effective evaluation of post-treatment cerebral aneurysms [8,9,10,11,12,13]. We aimed to evaluate the diagnostic value of IV CBCT in the assessment of post-treatment cerebral aneurysms when compared to MRA.

Preliminary results of this study had also been presented in the 47th Annual Meeting of European Society of Neuroradiology, Paris, France, 18–22 September 2024 [14].

## 2. Materials and Methods

### 2.1. Study Design

This is a single-center prospective cohort study performed at our hospital that aimed to evaluate the diagnostic value of IV CBCT in the assessment of post-treatment cerebral aneurysms.

This study was approved by the Research Ethics Committee.

Consecutive patients who had treatment for intracranial aneurysms with clinically scheduled MRA follow-up were recruited from July 2023 to April 2024.

Exclusion criteria are as follows:

- The presence of large or dense coil packing, as seen in those with giant aneurysm (aneurysm > 2.5 cm) or used for trapping.
- Patients with a modified Rankin score of 4 or 5.
- Illiterate and mentally incapacitated patients.
- Patients with severe chronic renal failure with an eGFR < 30 mL/min/1.73 m^2^ or those requiring renal replacement therapy.
- Patients with allergy to either iodinated or gadolinium contrast.

### 2.2. Imaging Protocol

Biplane DSA (Azurion FD20/15; Philips Healthcare, Best, The Netherlands) was used to perform IV CBCTA for the study patients. It consisted of a C-arm-mounted CT unit and a digital flat panel detector. Images were acquired with a delay of 18 s after intravenous contrast injection without the use of VasoCT. Images were created using a detector format of 22 cm, a 1016 × 1016 projection matrix without pixel binning (0.154 μm pixel pitch), 2586 × 1904 photodiodes, a scanning time of 21 s, 622 projections and an acquisition range of 240 degrees. The projections were acquired with 80 kV and 1820 mAs without Cu filtration at a source-to-image distance of 120 cm. The respective raw data were transferred to the Interventional Tools (Philips Healthcare) workstation for reconstruction. At a zoom factor of 100% and resolution of 384^3^, voxels of 273 × 273 × 273 µm were created. A second reconstruction on a specific area of interest was performed at 33% zoom and 512^3^ resolution. The isotropic volume data was displayed at a thickness of 0.27 mm. The metal artifact reduction algorithm (MARA) was utilized.

The contrast injection parameters for IV CBCT are as follows:

- Injection rate: 5 mL/s.
- Injection volume: 100 mL Omnipaque 350 (iodinated contrast medium) administered at full strength.

All MRA images were acquired on a 3T MAGNETOM Skyra scanner (Siemens Healthineers, HK) using a 20-channel head coil. Time-of-flight (TOF) MRA and contrast-enhanced (CE) MRA were performed for all patients (Table 1).

### 2.3. Qualitative Image Review

IV CBCTA and MRA images were anonymized and randomized for qualitative image review on two separate occasions by two independent neuroradiologists with 10 and 16 years of experience.

The diagnostic value of all MRA and IV CBCT datasets for each post-treatment intracranial aneurysm was evaluated across the following parameters using a 5-point Likert scale:

- Assessment of residual aneurysmal neck.
- Parent vessel status.
- Degree of artifacts of any form.

For those treated with stents, the following additional parameters were evaluated:

- Stent apposition to vessel wall.
- Delineation of stent struts.
- Vessel wall status of stented segment.

Image quality was scored on a 5-point Likert scale, as follows:

1. Unacceptable.
2. Poor.
3. Acceptable (acceptable for diagnostic use but with minor issues).
4. Good.
5. Excellent.

Image quality regarding the degree of artifacts was evaluated by a 5-point Likert scale:

1. Massive artifacts, significant distortion, parent vessel not differentiable, no diagnostic value.
2. Severe artifacts, moderate distortion, parent vessel poorly differentiable.
3. Moderate artifacts, mild distortion, satisfactory assessment of parent vessel status.
4. Mild artifacts without obvious distortion, parent vessel well differentiable.
5. Minimal or no artifacts.

A score of ≥3 was considered as an acceptable level of artifacts or as constituting adequate diagnostic acceptability.

During the image review, observers had the flexibility to adjust window-level settings and zoom on, pan and rotate images to optimize the image quality for assessment. Images were reviewed at axial, coronal and sagittal multiplanar volume-reformatted and 3D volume-rendered reconstructions.

A consensus in terms of the 5-point Likert scale was determined with the use of 10 cases that were excluded from the actual observer study.

### 2.4. Statistical Analysis

All statistical analyses were performed with SPSS (Version 29.0.1.0, 2023, IBM, Armonk, NY, USA.

Descriptive statistical analyses were performed on the variables of patient demographics.

Intra-observer and inter-observer agreement on the rating of the image quality of MRA compared with IV CBCT were estimated with weighted Cohen kappa (k), in which a k value of ≥0.8 signifies almost perfect agreement, and a k value of 0.6–0.79 indicates substantial agreement.

A two-tailed *p*-value less than 0.05 was considered statistically significant.

## 3. Results

A total of 97 consecutive patients who consented to participate in this study were scanned with both IV CBCT and MRA. Three patients were excluded as they had dense coil packing for giant aneurysm. A total of 114 aneurysms in 97 patients (mean age: 63.1 ± 11.7; 75 women and 22 men) were included. Furthermore, 9 out of 114 aneurysms were treated with surgical clipping, while 20 out of 114 aneurysms were treated solely with stents.

Overall, IV CBCT and MRA are comparable in terms of their assessment of parent vessel status and the degree of artifacts (*p* > 0.05) though MRA shows a slight advantage in evaluating residual aneurysmal neck (*p* = 0.05) (Table 2).

For clipped aneurysms, IV CBCT is superior in assessing residual aneurysmal neck (OR = 16.0, *p* < 0.001) and parent vessel status (OR = 15.1, *p* < 0.001) with significantly fewer artifacts (OR > 100, *p* < 0.001) (Table 3).

For aneurysms solely treated with stents, IV CBCT is superior in assessing residual aneurysmal neck (OR > 20, *p* = 0.002) and parent vessel status (OR > 20, *p* = 0.002) with significantly fewer artifacts (OR > 20, *p* = 0.002). IV CBCT outperforms MRA in evaluating stent struts and the vessel wall status of a stented segment when MRA is non-diagnostic (Table 4).

The overall inter-rater reliability is calculated as k > 0.7.

## 4. Illustrative Cases

### 4.1. Case 1

**Figure 1:**
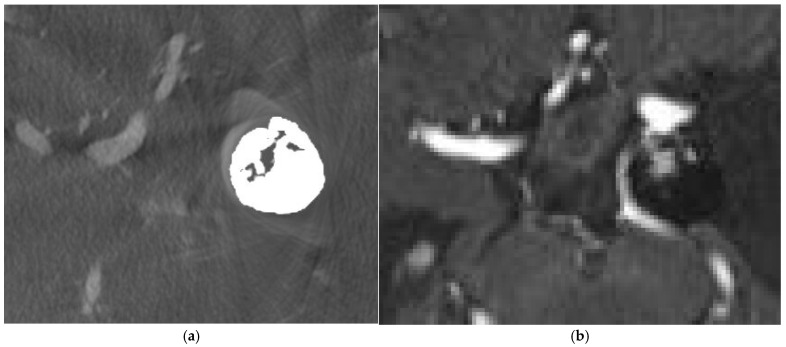
Coil embolization for left posterior communicating artery (PCoA) aneurysm. (**a**) IV CBCT with metallic artifact reduction (MAR) showed coil mass in left PCoA aneurysm with areas of loose packing or coil impaction, but no definite contrast enhancement was observed within aneurysmal sac. (**b**) Contrast-enhanced MRA showed contrast enhancement within coiled left PCoA aneurysmal sac, suggestive of slow residual flow.

A 80-year-old lady had coil embolization for ruptured left posterior communicating artery (PCoA) aneurysm 2 years ago. Follow-up IV CBCT and MRA were performed. (Figure 1)

### 4.2. Case 2

**Figure 2:**
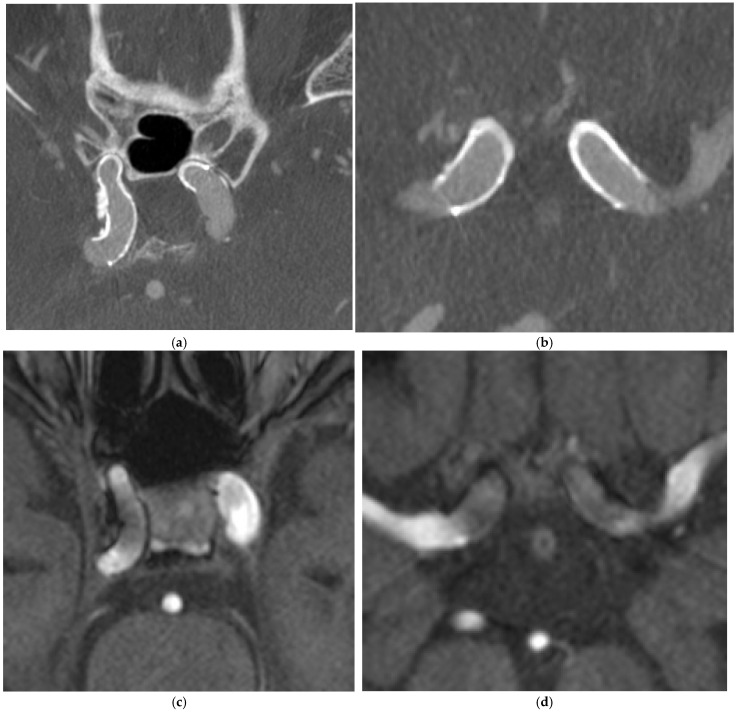
Flow diverter deployment for bilateral ophthalmic internal carotid artery (ICA) aneurysms. (**a**,**b**) IV CBCT with MAR showed stents well apposed to vessel wall, well-expanded stent ends and patent bilateral ICAs. Wall calcifications along stented segment of right ICA are also evident. (**c**,**d**) MRA showed bilateral stents with susceptibility artifacts. Stent apposition, stent struts, patency of parent vessel and vessel wall status of stented segment are compromised.

A 69-year-old lady had flow diverter deployment for bilateral unruptured ophthalmic internal carotid artery (ICA) aneurysms 4 years ago. IV CBCT and MRA were performed. (Figure 2)

### 4.3. Case 3

**Figure 3:**
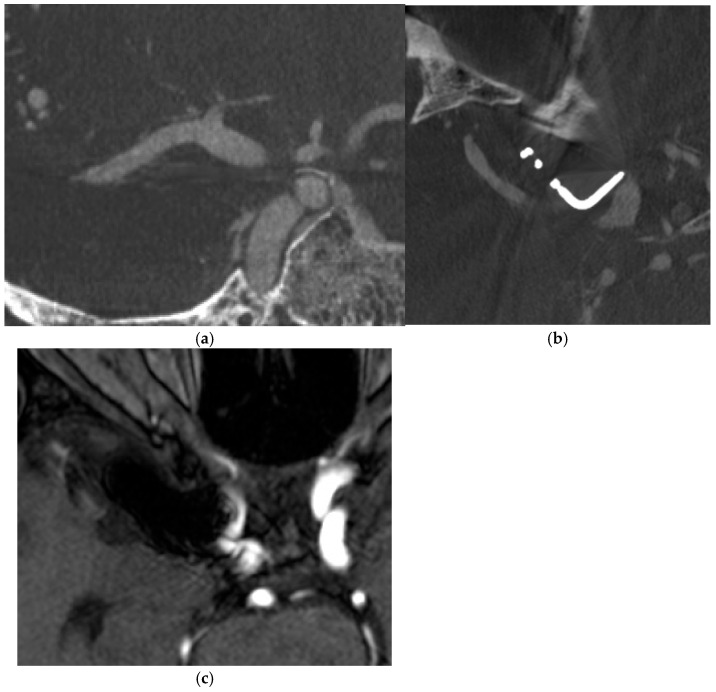
Clipping of right terminal internal carotid artery (ICA) aneurysm. (**a**,**b**) IV CBCT with MAR delineated clip well with no residual aneurysm and patent right internal carotid artery (ICA). (**c**) MRA demonstrates clip leading to marked susceptibility artifacts which render assessment impossible.

A 59-year old lady had clipping of ruptured right terminal internal carotid artery (ICA) aneurysm 15 years ago. Follow-up IV CBCT and MRA were performed. (Figure 3)

## 5. Discussion

In our institution, the first follow-up imaging is scheduled at 6 months post-treatment, with subsequent follow-ups at 6- to 12-month intervals depending on the findings from the initial post-treatment scans.

While digital subtraction angiography remains the gold standard for the surveillance of post-treatment intracranial aneurysms, it is impractical to utilize this as a long-term follow-up. Noninvasive imaging techniques including CT and MR cerebral angiogram are common alternatives. IV CBCT is another valuable noninvasive tool utilized for the same purpose [15].

IV CBCT with MARA effectively minimizes artifacts around metallic implants including coils, clips and stents for intracranial aneurysm treatment and allows for pronounced improvement for the delineation of surrounding structures, overcoming the limitations of multidetector CT cerebral angiogram [10,16,17].

Compared with multidetector row CT, CBCT also provides superior spatial resolution, which is distinctly beneficial for imaging intracranial vessels, especially those with metallic implants [15].

Moreover, the radiation dose of CBCT is lower than that of multidetector row CT as well [15].

Our findings indicate that IV CBCT is particularly beneficial for patients who have undergone solely clipping or stenting for intracranial aneurysms. The detailed structures of the surgical clip and stent could be clearly visualized without compromising the assessment of the parent vessel. For the surveillance of intracranial stents, IV CBCT facilitates the assessment of the patency of the parent vessel, the apposition of the stent to the vessel wall and vessel wall status, including stent-induced vascular calcifications. This further consolidates the findings from prior studies [8,9,10,11,12,13]. MRA, on the other hand, is limited in its assessment of these components due to susceptibility artifacts.

However, for coiled intracranial aneurysms, significant metallic artifacts in IV CBCT hinder the identification of tiny residual aneurysmal necks detected in MRA. Additionally, slow contrast filling within the coil mass detected in MRA [7] cannot be assessed in IV CBCT. Dense coil packing and large coil mass pose challenges in delineating tiny residual aneurysms. Nonetheless, the clinical significance of these tiny residual aneurysms detected only in MRA requires further evaluation. From our cohort, the smallest size of residual aneurysm that could be detected by IV CBCT is 1.5 mm.

Another limitation of CBCT is its limited ability to assess other intracranial pathologies, such as incidental intracranial or pituitary mass. MRI, with the option to utilize various sequences, can better accommodate the specific follow-up needs of patients with different conditions.

Recent technological advancements in CBCT hold promise for reducing metallic artifacts further and detecting even more tiny residual aneurysms than we observed in this cohort. The assessment of intracranial pathologies has also improved. Recent experts have proposed a novel dual-axis “butterfly” trajectory which has shown reduced artifacts in both supratentorial and infratentorial regions, improved contrast with the brain parenchyma and cerebrospinal fluid space and enhanced gray–white matter discrimination ability [18,19].

## 6. Limitations

We gathered data exclusively from one institution, exposing us to biases in the patient population available at our institution.

Our recruitment process focused on individuals who had clinically scheduled MRA, which could have potential selection bias.

Additionally, blinding was not possible, which could further contribute to bias.

A small sample size with 9 aneurysms treated solely with clips and 20 aneurysms treated solely with stents may introduce bias into the results.

The subjective assessment of image quality is also a potential bias.

## 7. Conclusions

IV CBCT and MRA have their own strengths and roles in the follow-up of post-treatment intracranial aneurysms. Overall, IV CBCT is superior in terms of its assessment of intracranial aneurysms treated solely with stents or surgical clips.

## Figures and Tables

**Table 1:** Scanning parameters for TOF MRA and CE MRA.

	TOF MRA	CE MRA
TR (ms)	24	20
TE (ms)	3.43	2.95
Flip angle	18	18
Acquisition matrix	319 × 384	319 × 384
Field of view	200 mm	200 mm
Slice thickness	0.8 mm	0.8 mm
Total acquisition time	2 min and 54 s	3 min and 1 s

**Table 2:** Overall observer rating on diagnostic value of IV CBCT and MRA.

	CBCT				MRA			
Assessment Parameters	Overall Agreement, %	Score 1–2 Agreement, %	Score ≥ 3 Agreement, %	k	Overall Agreement, %	Score 1–2 Agreement, %	Score ≥ 3 Agreement, %	k
Residual aneurysmal neck OR = 0.4 95% CI = 0.2–1.0 *p* = 0.05	75.4	15.8	83.3	0.803	82.5	7.0	92.1	0.791
Parent vessel status OR = 0.9 95% CI = 0.3–2.3 *p* = 0.79	80.7	9.6	84.2	0.821	75.4	8.8	87.7	0.755
Degree of artifacts OR = 1.2 95%CI = 0.5–2.9 *p* = 0.689	73.7	9.6	80.7	0.723	71.1	12.3	86.0	0.734
Stent apposition to vessel wall OR = 10.0 95% CI = 4.3–23.4 *p* < 0.001	75.6	7.3	87.8	0.781	82.9	43.9	51.2	0.834
Stent strut delineation OR > 1000 *p* < 0.001	82.9	14.6	80.5	0.833	100	100	0	1.0
Vessel wall status of stented segment OR > 1000 *p* < 0.001	78.0	14.6	82.9	0.805	95.1	100	0	0.828

**Table 3:** Observer rating on diagnostic value of IV CBCT and MRA on aneurysms treated with surgical clips.

	CBCT (02 vs. 03)				MRA (Q1–3: 01 vs. 03; Q4–6: 02 vs. 03)			
Assessment Parameters	Overall Agreement, %	Score 1–2 Agreement, %	Score ≥ 3 Agreement, %	k	Overall Agreement, %	Score 1–2 Agreement, %	Score ≥ 3 Agreement, %	k
Residual aneurysmal neck OR = 16.0 95% CI = 7.6–34.0 *p* < 0.001	77.8	11.1	88.9	0.775	88.9	66.7	33.3	0.800
Parent vessel status OR = 15.1 95% CI = 6.7–33.6 *p* < 0.001	55.6	11.1	55.6	0.595	77.8	66.7	22.2	0.804
Degree of artifacts OR > 100 *p* < 0.001	44.4	22.2	55.6	0.372	77.8	77.8	0	0.455

**Table 4:** Observer rating on diagnostic value of IV CBCT and MRA on aneurysms solely treated with stents.

	CBCT (02 vs. 03)				MRA (Q1–3: 01 vs. 03; Q4–6: 02 vs. 03)			
Assessment Parameters	Overall Agreement, %	Score 1–2 Agreement, %	Score ≥ 3 Agreement, %	k	Overall Agreement, %	Score 1–2 Agreement, %	Score ≥ 3 Agreement, %	k
Residual aneurysmal neck OR > 20 *p* = 0.002	95.0	0	100	0.857	70.0	10.0	90.0	0.613
Parent vessel status OR > 20 *p* = 0.002	90.0	0	100	0.783	65.0	10.0	90.0	0.565
Degree of artifacts OR > 20 *p* = 0.002	85.0	0	100	0.736	70.0	10.0	90.0	0.630
Stent apposition to vessel wall OR > 100 *p* < 0.001	85.0	0	100	0.348	75.0	35.0	55.0	0.734
Stent strut delineation	75.0	5.0	85.0	0.606	100	100	0	1.0
Vessel wall status of stented segment	75.0	0	100	0.464	95.0	100	0	0.857

Note: Overall agreement is determined when both observers provide identical scores. For each observer question, the k (weighted Cohen kappa) values, *p*-value, odds ratio (OR) and 95% confidence intervals (CIs) are given when applicable. The OR represents the improvement of classification from 1–2 to ≥3 with IV CBCT.

## Data Availability

The data presented in this study are available on request from the corresponding author due to privacy.
